# Magnesium-Induced Cell Survival Is Dependent on TRPM7 Expression and Function

**DOI:** 10.1007/s12035-019-01713-7

**Published:** 2019-08-08

**Authors:** Yuyang Sun, Pramod Sukumaran, Brij B. Singh

**Affiliations:** Department of Periodontics, School of Dentistry, University of Texas Health Science Center San Antonio, San Antonio, TX 78229 USA

**Keywords:** TRPM7, Mg^2+^ homeostasis, Caspase activity, Cell death and neurodegeneration

## Abstract

Mg^2+^ homeostasis is essential for cell survival and the loss of this regulation has been associated with many neurodegenerative diseases, including loss of dopaminergic neurons. Although the neurotoxin-mediated loss of dopaminergic neurons in Parkinson disease models is extensively studied, the ion channel(s) that regulate Mg^2+^ homeostasis and thus could prevent neuronal cell death is not yet identified. Here, we show that TRPM7 (transient receptor potential melastatin 7) is involved in regulating Mg^2+^ homeostasis in dopaminergic cells. Importantly, transient loss of TRPM7 decreased intracellular Mg^2+^ levels and decreased dopaminergic cells/neurons survival. We provide further evidence that both increases in extracellular Mg^2+^ or transiently increasing TRPM7 levels protected dopaminergic SH-SY5Y cells against neurotoxin-mediated cell death. Neurotoxin treatment significantly decreased TRPM7 levels in both SH-SY5Y cells and the substantia nigra pars compacta region of mice, along with a decrease in Mg^2+^ influx. Moreover, Mg^2+^ supplementation showed a concentration-dependent decrease in caspase-3 activity, an increase in cell survival, restored mitochondrial membrane potential, and increase TRPM7 levels in neurotoxin-treated cells. In contrast, transient silencing of TRPM7 inhibited the positive effect of Mg^2+^ supplementation in protecting against neurotoxins. Whereas, TRPM7 overexpression not only maintained Mg^2+^ homeostasis but also inhibited caspase 3 activity that induced cell survival. Overall, these results suggest a significant role of TRPM7 channels in Mg^2+^ homeostasis and the survival of neurotoxin-induced loss of dopaminergic cells.

**Electronic supplementary material:**

The online version of this article (10.1007/s12035-019-01713-7) contains supplementary material, which is available to authorized users.

## Introduction

Parkinson’s disease (PD) is a common neurodegenerative disorder and loss of dopaminergic (DA) neurons in the substantia nigra pars compacta region (SNpc) underlies the main motor symptoms observed in PD [1]. Additionally, non-motor symptoms that include cognitive impairment, as well as autonomic, olfactory, sleep, mood disorders, and gut physiology, are also observed upon the loss of DA neurons. Several factors such as aging, exposure to various neurotoxins, and inflammation lead to the vulnerability of DA neurons that could induce PD. Importantly, protection against toxins or prevention of the loss of DA neurons has been identified as a possible therapeutic mechanism to prevent/treat PD. Recent studies have suggested that imbalance in divalent cations, ER stress, oxidative stress, and/or mitochondrial dysfunction is an important mechanism in the pathogenesis of PD [2–5]. Interestingly, the role of divalent cations especially magnesium (Mg^2+^) in neuron survival has gained much attention in recent years [6]. Mg^2+^ is also fundamental in many cellular processes including cell proliferation and has been shown to modulate physiological functions, such as mitochondrial respiration, synthesis of biomacromolecules, and energy metabolism, which are essential for neuronal survival [7, 8]. Importantly, appropriate intracellular Mg^2+^ levels also modulate nucleic acid metabolism, protein synthesis, and energy production [9, 10]. Moreover, it has been proposed that alterations in ion (Mg^2+^ and Ca^2+^) homeostasis are also involved in the pathogenesis of PD [11–13]. Magnesium-deficient mice exhibit enhanced susceptibility to MPTP-mediated neurotoxicity in DA neurons [14]. Rats treated with 6-hydroxydopamine (a common neurotoxin-induced PD animal model) had lower levels of intracellular Mg^2+^ compared with control untreated mice. In addition, regional Mg^2+^ concentrations are decreased in samples of PD patients, which is strongly correlated with the duration and the severity of the disease [11, 12], but the ion channel(s) responsible for maintaining Mg^2+^ homeostasis in dopaminergic cells is not known.

Among several ion channels present in mammalian cells, melastatin-like transient receptor potential 6 and 7 (TRPM 6 and 7) channels have been shown to conduct Mg^2+^ at negative membrane potentials [15]. Similarly, the solute carrier family 41, member 1 (SLC41A1) has also been shown to be involved in Mg^2+^ transport and SLC41A1 is a candidate for the causative gene in the PARK16 locus, where variation is associated with risk for idiopathic PD [16]. Unlike SLC41A1, TRPM7 channels are widely expressed and support multiple cellular and physiological functions, including cellular Mg^2+^ homeostasis, growth/cell viability, neuronal cell death, synaptic transmission, and adhesion [17, 18]. We have previously shown that in non-excitable cells, TRPM7 is tightly regulated with cellular Mg^2+^ concentration [19]; however, its role in neurotoxin models of PD is not well studied. Neurodegeneration comprises the assembly of several pathological events that could lead to a progressive loss of neuronal structure and function including cellular damage, disease development, or cellular death [20]. Importantly, most of these processes are dependent on Mg^2+^ and as TRPM7 regulates Mg^2+^ homeostasis, it could be postulated that TRPM7 would play a major role in these conditions. Interestingly, a missense variant of *TRPM7* (T1482I) has been reported in Guamanian ALS/PD patients [21]. However, another study failed to observe similar mutations in TRPM7 channels [22]. Similarly, rats that were exposed to restricted intake of Ca^2+^ and/or Mg^2+^ over two generations resulted in severe loss of DA neurons [13]. In addition, mutations in TRPM7 also resulted in a defect in DA neurons in zebrafish models [23]. Together, these studies suggest that Mg^2+^ homeostasis via TRPM7 could play a key role in neuronal function [24]. However, its expression and its role in the degeneration of DA neurons/cells have not yet been identified.

The purpose of the current study was to establish the role of TRPM7 in neurotoxin-induced DA cell death and understand the mechanism as to how Mg^2+^ supplementation could protect DA neurons. The data presented here demonstrate that Mg^2+^ supplementation inhibits neurotoxin-induced apoptosis which is dependent on TRPM7 expression and function. Our data also shows that TRPM7 is a major Mg^2+^ channel in DA neurons and its expression and function are decreased upon neurotoxin treatment. The knockdown of TRPM7 abolished the protective effect associated with Mg^2+^. Furthermore, TRPM7 overexpression prevented MPP^+^-induced cellular death by inhibiting apoptosis in neuronal cells, whereas inhibiting TRPM7 function decreased Mg^2+^ influx and increased neuronal loss. Together, these results suggest that TRPM7 could play an important role in protecting DA cells from neurotoxin-induced degeneration.

## Materials and Methods

### Cell Culture, Transient Transfections, and Viability Assays

SH-SY5Y cells were obtained from the American Type Culture Collection (Manassas, VA) and cultured/maintained at 37 °C with 95% humidified air and 5% CO_2_ and differentiated as previously described [25]. For different level Mg^2+^ experiment, customized culture media without Mg^2+^ are obtained from Gibco and different level of MgCl_2_ was added as required for individual experiments. For transient transfection experiments, shRNA plasmid that targets the coding sequence of human TRPM7 was obtained from Origene (Rockville, MD) and transfected using lipofectamine. Similarly, for transient overexpression of HA-TRPM7, a plasmid expressing full-length TRPM7 (cloned in pcDNA3 using a CMV promoter) was used and 5 μg of plasmid DNA was transformed using lipofectamine in Opti-MEM medium as per supplier’s instructions and assayed after 24 or 48 h as indicated in the figures. For cell viability assays, cells were counted and seeded equally on 96-well plates at a density of 0.5 × 10^5^ cells/well. The cultures were grown for 24 h under different conditions and cell viability was measured by using the MTT method as described previously [2]. Cell viability was expressed as a percentage of the control (untreated) when compared with neurotoxin treatment.

### Animals and Human Brain Samples

Six-month-old male C57BL/6 mice (Charles River, USA) /were used for these experiments. All animals were housed in a temperature-controlled room under a 12-/12-h light/dark cycle with ad libitum access to food and water and experiments were carried out as per the institutional guidelines (protocol approved by IACUC committee) for the use and care of animals. For Mg^2+^ supplement group, water containing 900 mg/kg/day of magnesium aspartate/mice daily was given ad libitum for 4 weeks and water consumption was also measured daily. For PD mice model, 6-month-old mice were challenged with either MPTP (MPTP-HCl, 25 mg/kg per injection, i.p.) or saline for 6 consecutive days at 24-h intervals. Mice were sacrificed 7 days after the last MPTP injection and substantia nigra was isolated. TH staining was performed on various sections as described previously by us [2]. Postmortem human substantia nigra samples of control (6 samples, males with age ranging from 65 to 72) and PD patients (8 samples, males/females ages 60–72, with severe motor neuron deficit) were immediately frozen and obtained from UK Parkinson’s foundation. All human subjects were approved by the institutional IRB and that the studies abide by the Declaration of Helsinki principles.

### Electrophysiology

For patch clamp experiments, coverslips with cells were transferred to the recording chamber and perfused with an external Ringer’s solution of the following composition (mM): NaCl, 145; CsCl, 5; MgCl_2_, 1; CaCl2, 1; Hepes, 10; Glucose, 10; pH 7.3 (NaOH). The MgCl_2_ concentration was adjusted depending on the conditions (1 mm or 10 mM); similarly, NH_4_Cl was included in the bath solution. Whole-cell currents were recorded using an Axopatch 200B (Axon Instruments, Inc.). The patch pipette had resistances between 3 and 5 MΩ after filling with the standard intracellular solution that contained the following (mM): cesium methanesulfonate, 150; NaCl, 8; Hepes, 10; EDTA, 10; pH 7.2 (CsOH). With a holding potential 0 mV, voltage ramps ranging from – 100 to + 100 mV and 100-ms duration were delivered at 2-s intervals after whole-cell configuration was formed. Currents were recorded at 2 kHz and digitized at 5–8 kHz. pClamp 10.1 software was used for data acquisition and analysis. Basal leak currents were subtracted from the final currents (when current reach the peak) and average currents are shown. All experiments were carried out at room temperature and the data presented are from 6 to 10 individual cells patched for each condition.

### Magnesium/Calcium Imaging

Cells were incubated with 2 μM Mag-Fura 2-AM (Invitrogen) or Fura-2 (Molecular Probes for 45 min, washed twice with SES (Standard External Solution, includes 10 mM HEPES, 120 mM NaCl, 5.4 mM KCl, 1 mM MgCl_2_, 1 mM CaCl_2_, 10 mM glucose, pH 7.4) buffer. For fluorescence measurements, the fluorescence intensity of Fura-2-loaded control cells was monitored with a CCD camera–based imaging system mounted on an Olympus XL70 inverted microscope. Fluorescence traces shown represent [Mg^2+^]_i_ values that are averages from at least 30–40 cells and are a representative of results obtained in at least 3–4 individual experiments. Relative Mg^2+^ or Ca^2+^ concentrations in individual cells were estimated from the 340-/380-nm ratio.

### Western Blot Analysis

Radioimmunoprecipitation assay (RIPA) buffer was used to obtain cell lysates from both SH-SY5Y cells, and SNpc region of the brain. Protein concentrations were determined, using the Bradford reagent (Bio-Rad), and 25 μg of lysates were resolved on NuPAGE 4–12% Bis-Tris gels (Invitrogen, Carlsbad, CA), followed by Western blotting as described [25, 26], using the following monoclonal or polyclonal antibodies: anti-TRPM7 (Abcam, MA; Cat# 109438; Dilution in 1:500), anti-LC3A (Cell Signaling, MA; Cat# 4599; Dilution in 1:1000), anti-Bcl2 (Cell Signaling, MA; Cat# 209039; Dilution 1:1000), anti-Bax (Cell Signaling, MA; Cat# 5023; Dilution used was 1:1000), anti-Caspase 3 (Cell Signaling, MA; Cat# 9662; Dilution used was 1:2000), anti-β-Actin (Cell Signaling, MA; Cat# 4970; at 1:2000 dilution).

### Mitochondrial Membrane Potential and Caspase 3 Activity

The mitochondrial transmembrane potential was also measured using fluorescent cationic dye rhodamine 123 as described [26]. The fluorescence signals of the dye were measured immediately at an excitation wavelength of 488 nm and an emission wavelength of 510 nm using a fluorescence microplate reader. The results were expressed as percentage of an increase or decrease in fluorescence above the control fluorescence. Caspase 3 activity was measured using abcam Caspase 3 assay kit. One million cells were isolated using cell lysis buffer and the liquid fraction was used to analyze the caspase 3 activity as manufacturer’s instructions. The sample absorbance was measured at 450 nm and the graph was plotted using absorbance value.

### Statistical Analysis

Data analysis was performed using Origin 9.0 (Origin Lab). Statistical comparisons were made using Student’s *T* test or one-way ANOVA (post hoc using Tukey or Fisher test). Experimental values are expressed as means ± SEM or ± SD along with individual data points. Differences in the mean values were significant at *p* value < 0.05.

## Results

### Neurotoxin-Induced Loss of DA Neurons Are Protected upon Magnesium Supplementation

**Fig. 1:**
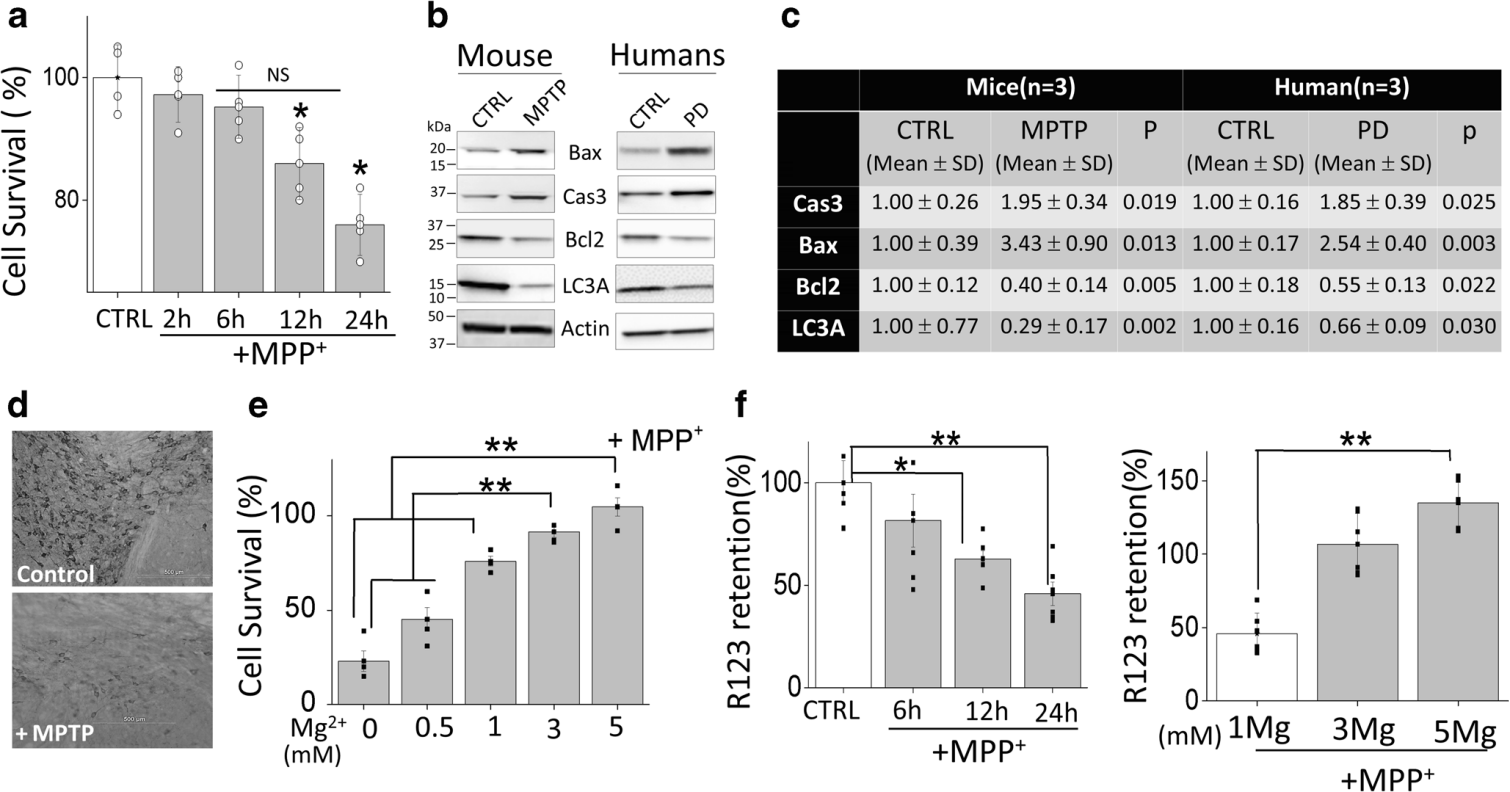
Magnesium supplement rescued the neurotoxin-induced loss of dopaminergic cells: **a** MTT assays performed on control and MPP^+^ (500 μM) treatment SH-SY5Ycells at different time points. The columns show the means ± SD of 5 individual experiments performed in triplicate. A one-way ANOVA was conducted to compare the effect of neurotoxin treatment (time-dependent) on cell survival (*p < 0.05, **p < 0.01; one-way ANOVA, Tukey post hoc test). **b** and **c** Western blots showing the expression of Bax, Caspase 3, Bcl2, LC3A, and loading control β-actin in SNpc tissues from control and MPTP treatment mice, or from control and PD samples, were resolved and analyzed by western blotting. Antibodies used are labeled in the figure. Quantification of the blots is shown in table (two-tailed unpaired *T* test). **d** A representative image of the expression of TH-positive neurons in the SNpc region of control and MPTP-treated mice. **e** MTT assays showing the effect of Mg^2+^ supplement (application with MPP^+^ at the same time) the survival of neuroblastoma cells. The columns show the means ± SD of 4 experiments normalized with MPP^+^ untreatment cells. (**p < 0.01; one-way ANOVA, Tukey post hoc test). **f** Mitochondrial membrane potential was evaluated using the fluorescent cationic dye Rh123 under various conditions as labeled in the figure. The columns show the means ± SEM of 6 independent experiments performed in duplicate. (*p < 0.05, **p < 0.01; one-way ANOVA, Tukey post hoc test)

Neurotoxins, 1-methyl-4-phenyl-1,2,3,6-tetrahydropyridine (MPTP), have been shown to selectively degenerate DA neurons in humans, sub-human primates, and lower animals that mimic PD-like symptoms [27]. MPTP is converted in the brain into MPP^+^ and selectively taken up by DA neurons, which is concentrated within the mitochondria and decreases mitochondrial membrane potential thereby initiating neuronal loss [28, 29]. We first examined the effect of MPP^+^ on the survival of DA cells. MPP^+^ treatment showed a time-dependent increase in cell death in differentiated SH-SY5Y cells (Fig. 1a), which is consistent with previous reports [29]. Importantly, comparisons using the Tukey post hoc test indicated that the mean score for neurotoxin treatment at 12 or 24 h was (*M* = 86.00, 76.00, SD = 5.89, 4.97, respectively), which was significantly lower than untreated control conditions. In comparison with control, no significant change in cell survival was observed at 2 or 6 h of neurotoxin treatment. Consistent with cell line data, MPTP treatments also significantly increased expression of the apoptotic protease caspase 3 and pro-apoptotic proteins Bax and decreased expression of Bcl_2_ in the SNpc region (Fig. 1b, c). Similar results were also observed in samples obtained from the SNpc region of human PD patients where expression of apoptotic proteins (caspase 3, Bax, and Bcl2) was increased when compared with age-matched control human samples (Fig. 1b, c).

Recent studies have shown that loss of autophagy could induce apoptosis in neurodegenerative diseases [3, 30]. Hence, we also evaluated the expression of LC3A, an autophagic marker, and a significant decrease in LC3 expression was observed in samples obtained from PD patients and neurotoxin-treated SNpc samples (Fig. 1b, c). We next evaluated the presence of TH-positive neurons in the SNpc region, which again showed a decrease in the total number of TH-positive neurons in the SNpc region that were subjected to neurotoxin (Fig. 1d). These results are consistent with our previous studies [2, 3] and also establish that loss of autophagy and activation of caspase 3, in samples from PD patients and neurotoxin models, might be the reason for the loss of these TH-positive neurons. Importantly, increasing Mg^2+^ concentration in SH-SY5Y cells significantly rescued MPP^+^-induced cell death and protection from neurotoxins was only observed at 3 or 5 mM Mg^2+^, which was significantly higher than at lower Mg^2+^ (0, 0.5, 1 mM) concentrations (Fig. 1e). Taken together, these results suggest that Mg^2+^ levels do have a positive effect on neurotoxin-induced cells death. However, it should be noted that higher Mg^2+^ levels were needed for the protection of neuroblastoma cells from MPP^+^. As mitochondrial membrane potential is critical, we used rhodamine 123 to elucidate the role of Mg^2+^ supplementation in regulating neurotoxin-mediated loss of mitochondrial membrane potential. As expected, 12-h and 24-h MPP^+^ treatments resulted in a reduction of mitochondrial membrane potential as compared with control untreated cells (post hoc comparison showed that *M* = 62.93, 45.94, SD = 9.55, 14.00, respectively), which was significantly different than untreated cells (*M* = 100.01, SD = 26.32) (Fig. 1f). An increase in Mg^2+^ concentration again showed restoration of mitochondrial membrane potential, even in the presence of MPP^+^. Importantly, intracellular Mg^2+^ levels were also increased upon supplementation with higher external Mg^2+^ concentrations (3 or 5 mM) (supplemental Fig. 1A-D) Together, the results presented here suggest that Mg^2+^ supplementation restores MPP^+^-mediated loss in mitochondrial membrane potential and rescues MPP^+^-mediated cell death in neuroblastoma cells.

### Magnesium Supplement Inhibits Neurotoxin-Induced Apoptosis to Promote Autophagy

**Fig. 2:**
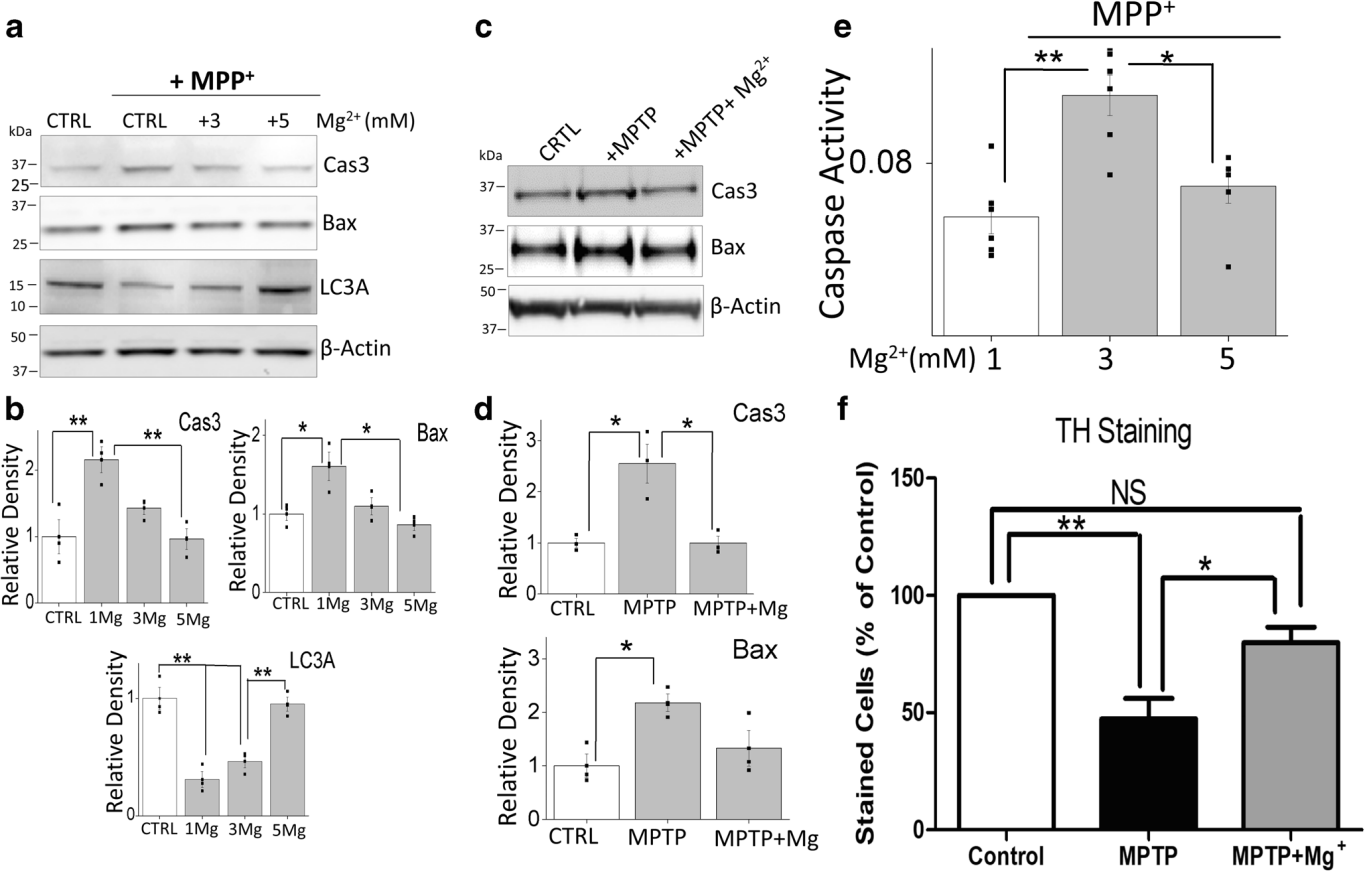
Magnesium supplement decreased neurotoxin-induced apoptosis: **a** Western blots showing the expression of Bax, Caspase 3, Bcl2, LC3A, and loading control β-actin in CTRL and MPP^+^-treated (500 μM) cells for 24 h with different magnesium concentrations in SH-SY5Y cells. Quantification of the blots is shown as bars in (**b**) and shows the means ± SEM of 3 experiments. An asterisk indicates significance than control (*p < 0.05, **p < 0.01; one-way ANOVA, Tukey post hoc test). **c** Caspase 3 activity was evaluated under various conditions in SH-SY5Y cells. The columns show the means ± SEM of 5 independent experiments (performed in duplicate). (*p < 0.05, **p < 0.01; one-way ANOVA, Tukey post hoc test). **d** SNpc tissues from control and MPTP treatment with and without Mg^2+^ supplement mice were resolved and proteins as labeled, were analyzed by western blotting. **e** Quantification of western blots, representing the means ± SEM of 3 independent experiments performed in duplicate, are shown. (*p < 0.05; one-way ANOVA, Tukey post hoc test). **f** Quantification of the TH-positive neurons in various conditions is shown as bar graph and represents the means ± SEM of 3 independent experiments. A single and double asterisk indicates significance than control (*p* < 0.05, *p* < 0.01 respectively using the ANOVA, Tukey Test)

To understand the role of Mg^2+^ in neurotoxin-induced apoptosis, we first examined the expression of apoptosis-related proteins in SH-SY5Y cells. MPP^+^ treatment showed an increase in the expression of the apoptotic protease caspase 3 and Bax in regular media (CTRL that has 1 mM Mg^2+^) (Fig. 2a, b). However, increasing Mg^2+^ concentration (3 or 5 mM) significantly decreased the expression of apoptotic proteins Bax and caspase 3 (Fig. 2a, b). In addition, MPP^+^ also induced a loss of expression of autophagic marker LC3A, which was again significantly reversed by increasing the external Mg^2+^ concentration (maximum protection was observed at 5 mM) (Fig. 2a, b). Moreover, caspase 3 activity was also increased in cells that were treated with MPP^+^ and a subsequent increase in Mg^2+^ concentration significantly inhibited the caspase 3 activity when compared with untreated controls (Fig. 2c). Similar results were observed in the mice SNpc region where MPTP treatment also showed increased activation of apoptotic proteins (Bax and caspase 3), which were again decreased upon Mg^2+^ supplementation (Fig. 2d, e). Importantly, the supplementation of Mg^2+^ in mice that were treated with MPTP also showed significant protection as observed by the loss of TH-positive neurons in the SNpc region (Fig. 2f). Cumulatively, these results suggest that Mg^2+^ might play an important role in the neurotoxin-induced apoptosis and loss of TH-positive dopaminergic neurons.

### TRPM7 Expression and Function Are Decreased Under MPP^+^ Treatment in SH-SY5Y Cells

**Fig. 3:**
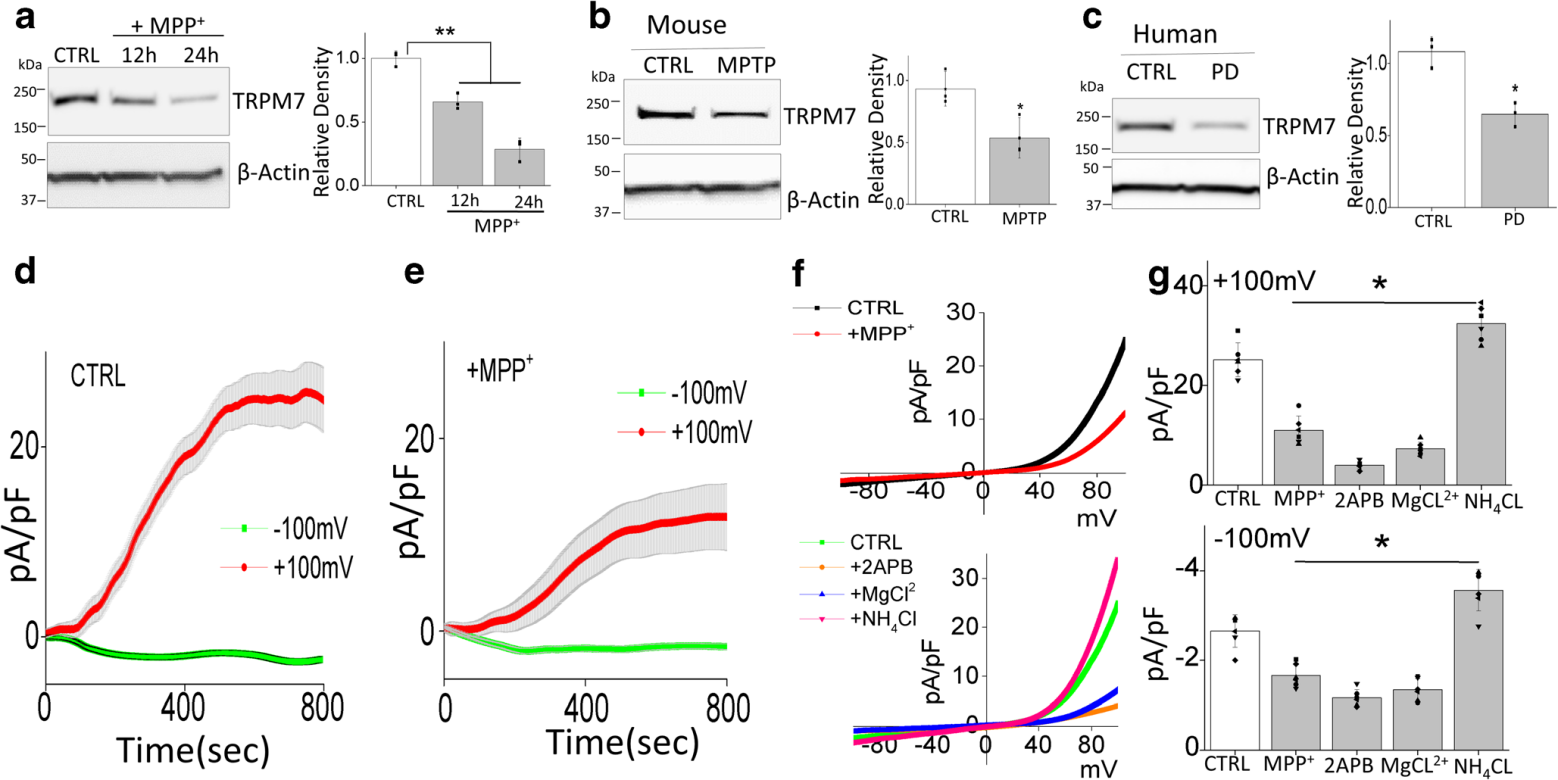
TRPM7 expression and function are decreased upon MPP^+^ treatment: Sample from SH-SY5Y (**a**), mice (**b**), and PD patients (**c**) were resolved and TRPM7 expression was analyzed by western blotting. The columns show the means ± SD of 3 experiments. (*p < 0.05, **p < 0.01); **a** One-way ANOVA, Tukey post hoc test; **b** and **c** Two-tailed unpaired *T* test. Individual traces from whole-cell recording in normal SES (1 mM Ca^2+^, 1 mM Mg^2+^) bath solution showing outward/inward currents at + 100 mV/− 100 mV in control (**d**) and MPP^+^ (**e**)-treated (500 μM, 24 h) SH-SY5Ycells are presented. IV curves (acquired when currents reach peak) under various conditions (pre application 500 μM 2APB, 10 mM MgCl_2_, or 10 mM NH_4_CL for 5 min before recording) as labeled in the figure are shown in (**f**) and quantitation of current density at ± 100 mV is shown in (**g**). The columns show the means ± SD of 6 experiments. (**p < 0.01; one-way ANOVA, Tukey post hoc test)

We next evaluated the ion channel essential for Mg^2+^ homeostasis and as TRPM7 is an important ion channel related to Mg^2+^ transport, we investigated the relationship of TRPM7 and neurotoxin treatment. Addition of MPP^+^ or MPTP treatments significantly decreased TRPM7 level in vitro (Fig. 3a) and in vivo (Fig. 3b) respectively. Also, TRPM7 levels were downregulated in SNpc region of human PD patients (Fig. 3c), when compared with age-matched control samples. To establish that TRPM7 is functional in dopaminergic cells, whole-cell current recordings were performed in differentiated SH-SY5Y cells. An inward and outward rectifying currents that reversed around 0 mV were observed in SH-SY5Y cells, which was decreased in neurotoxin-treated cells (Fig. 3d–g). To investigate the pharmacological properties of these currents, we next studied the effects of 2-APB. 2APB has been shown to inhibit TRPM7 function but potentiates TRPM6 function [31, 32], which also exhibit similar electrophysiological properties. Addition of 2APB significantly inhibited both the inward and outward currents (Fig. 3f, g). Also, the current properties were consistent with the previous recording performed in other cells, which are linked with TRPM7 channels [31]. Moreover, another distinguishing characteristic of the TRPM7 channel is that it is sensitive to external Mg^2+^ concentrations, but is activated by the addition of NH_4_Cl. Importantly, the TRPM7 current was inhibited by bath application of 10 mM Mg^2+^ and facilitated in the presence of 10 mM NH_4_Cl (Fig. 3f, g), which were consistent with previous studies [33, 34] and further confirmed that the currents observed are indeed mediated through the TRPM7 channels in these cells. Importantly, MPP^+^ treatment had a prominent effect and a significant decrease in TRPM7 currents was observed in SH-SY5Y cells (Fig. 3D–G). These results demonstrate that TRPM7 expression and function were inhibited under neurotoxin treatment in dopaminergic cells which might contribute to their loss.

### Mg^2+^-Induced Protection Against Neurotoxin Was Dependent on TRPM7 Expression and Function

**Fig. 4:**
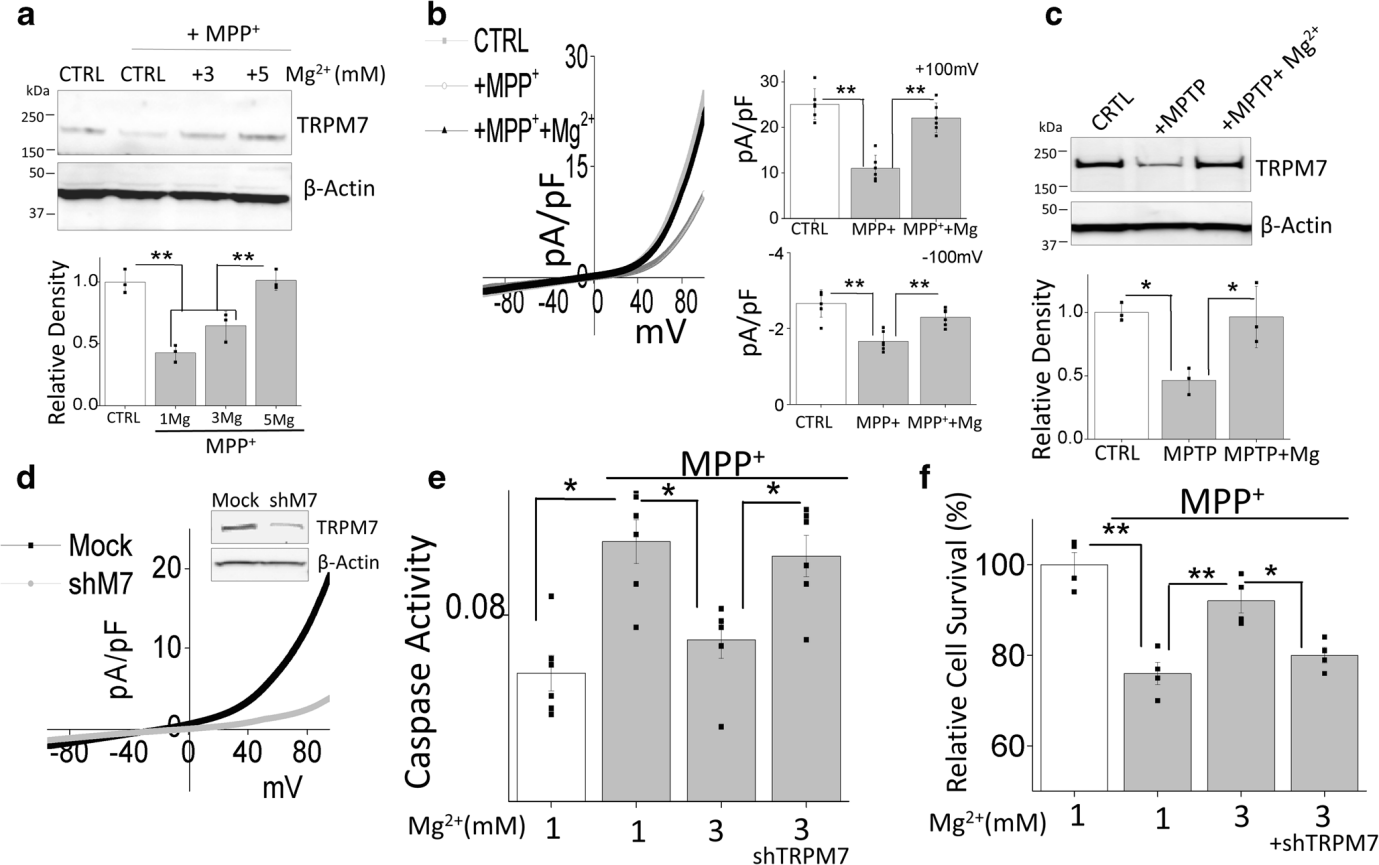
Neurotoxin-induced apoptosis in dopaminergic neurons is inhibited upon Mg^2+^ supplementation: **a** Western blots showing the expression of TRPM7 and loading control β-actin in CTRL and MPP^+^-treated (500 μM, 24 h) cells with different magnesium supplement in SH-SY5Y cells. The columns represent the mean ± SD of 3 independent experiments. (**p < 0.01; one-way ANOVA, Tukey post hoc test). **b** IV curve (acquired when currents reach peak) and quantitation of electrophysiological results in normal SES solution (1 mM Ca^2+^, 1 mM Mg^2+^) are shown from control, MPP^+^ treatment (500 μM, 24 h), and Mg^2+^ supplemented (5 mM, 24 h) SH-SY5Y cells. The columns show the means ± SD of 6 experiments. (**p < 0.01; one-way ANOVA, Tukey post hoc test). **c** SNpc tissues from control, MPTP treatment, and Mg^2+^ supplement mice were resolved and analyzed by western blotting. The columns presented are the mean ± SD of 3 experiments. (*p < 0.05; one-way ANOVA, Tukey post hoc test). **d** Western blotting and electrophysiological results (maximum peak currents) showing TRPM7 expression and function in mock or TRPM7silenced (transient expression of shM7 for 24 h) SH-SY5Y cells. **e** Caspase 3 activity was evaluated under various conditions in SH-SY5Y cells and bar graph columns represent mean ± SEM of 6 experiments. (*p < 0.05, **p < 0.01; one-way ANOVA, Tukey post hoc test). **f** MTT assays were evaluated under various conditions in SH-SY5Y cells. The columns show the means ± SEM of 4 experiments. (*p < 0.05, **p < 0.01; one-way ANOVA, Tukey post hoc test)

The results presented above demonstrate that Mg^2+^ supplement is essential for neuronal protection and TRPM7 channel might be involved. Hence, we directly investigated the relationship between TRPM7, Mg^2+^, and neurotoxin treatment. An increase in external Mg^2+^ concentration significantly restored TRPM7 expression that was decreased upon MPP^+^ treatment in SH-SY5Y cells (Fig. 4a). Furthermore, TRPM7 function was also recovered after increasing Mg^2+^ concentration (Fig. 4b) and increase in intracellular Mg^2+^, but not Ca^2+^, was also observed by increasing external Mg^2+^ (Supplemental Fig. 1A-D). Additionally, in vivo experiments also showed similar results and increase in TRPM7 expression was observed upon Mg^2+^ supplementation (Fig. 4c). To further understand the role of TRPM7 in protecting against MPP^+^-induced cell death, we transiently knocked down the expression of TRPM7 in SH-SY5Y cells. Silencing of TRPM7 decreased TRPM7 protein levels and a subsequent decrease in TRPM7 currents was observed (Fig. 4d). Importantly, knockdown of TRPM7 expression abolished the Mg^2+^-induced inhibition of the caspase 3 activity (Fig. 4e), indicating that inhibition of apoptosis by Mg^2+^ is dependent on TRPM7 expression and function. Similarly, the protective effect of Mg^2+^ on cell survival was also abolished with the knockdown of TRPM7 expression (Fig. 4f). Together, these results suggest that the protective effect of Mg^2+^ supplementation was dependent on TRPM7 expression.

### Overexpression of TRPM7 Protected Against MPP^+^-Induced Cell Death

**Fig. 5:**
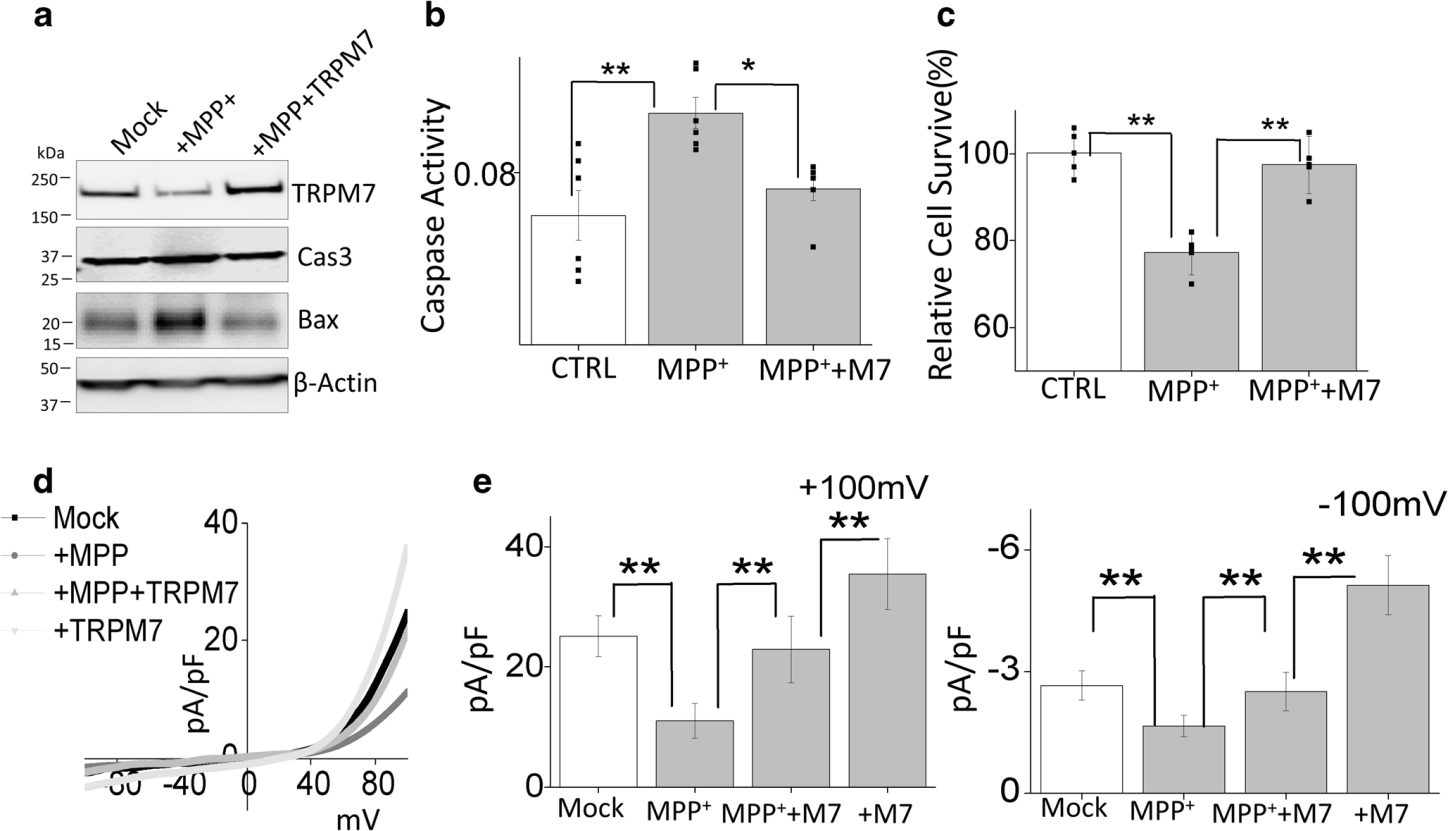
Overexpression of TRPM7 protected against MPP^+^-induced cell death: **a** Protein lysates from control, MPP^+^(500 μM, 24 h), and MPP^+^ + TRPM7 overexpressed (transient expression for 48 h) SH-SY5Y cells were resolved on SDS gels and analyzed by western blotting. **b** Caspase 3 activity was evaluated under similar conditions (as labeled in the figure) in SH-SY5Y cells. The columns show the means ± SEM of 4 separate experiments performed in duplicate (*p < 0.05, **p < 0.01; one-way ANOVA, Tukey post hoc test). **c** MTT assays were evaluated under various conditions in SH-SY5Y cells. The columns show the means ± SD of 4 independent experiments (**p < 0.01; one-way ANOVA, Tukey post hoc test). **d** IV curves (acquired when currents reach its peak) of TRPM7 currents under various conditions and quantitation of current density at ± 100 mV (mean ± D of 6 experiments) are shown in (**e**). (**p < 0.01; one-way ANOVA)

To finally establish the role of TRPM7 in the loss of dopaminergic cells, we transiently overexpress TRPM7 in differentiated SH-SY5Y cells (Fig. 5a). Overexpression of TRPM7 not only abolished MPP^+^-induced increase in the expression of apoptotic protease caspase 3, pro-apoptotic proteins Bax (Fig. 5a), but also decreased neurotoxin-induced increase in caspase 3 activity (Fig. 5b). In addition, overexpression of TRPM7 also significantly decreased MPP^+^-induced cell death (Fig. 5c). These results suggest that the expression of TRPM7 is essential for neuronal survival and neurotoxin-induced loss of TRPM7 could contribute towards the loss of dopaminergic neurons. Moreover, overexpression of TRPM7 significantly increased the TRPM7 currents and was able to restore the Mg^2+^ currents when compared with MPP^+^ treatment (Fig. 5d, e). Cumulatively, these results further confirmed that the protective effect of Mg^2+^ supplementation in neurotoxin-treated cells are dependent on TRPM7 expression and function, which inhibits the activation of pro-apoptotic proteins and limits neuronal loss.

## Discussion

By using neurotoxin, as a model for PD, we have demonstrated that Mg^2+^ homeostasis is essential for the inhibition of neurotoxin-induced cell death in DA cells, which is consistent with previous reports [14, 35]. Mitochondrial dysfunction is a typical feature that is observed in neurodegeneration including PD. Mutations in genes owing to mitochondrial quality control are the main reasons that lead to familial PD [36, 37]. In addition, mitochondrial activity linked to cellular ATP synthesis is one of the key regulators of neurotoxin-induced cell death. Mg^2+^ is an essential cation that modulates a number of physiological processes including mitochondrial function [38, 39]. Our data indicate that loss of Mg^2+^ homeostasis leads to a decrease in mitochondrial potential, which could prevent the translocation of Bax to mitochondria thereby inhibiting the activation of caspases that are essential for apoptosis [40]. Apoptosis has been shown as the main mechanism that initiates DA neuronal loss in PD. Upon MPTP induction, expression of pro-apoptotic protein Bax was increased, which could initiate mitochondrial-mediated cell death by sequestering anti-apoptotic members such as Bcl_2_, leading to the activation of caspases as observed here. Importantly, our data showed that Mg^2+^ supplementation significantly decreased Bax expression, reduced caspase 3 activation, and promoted cell survival. These results are important as they suggest that Mg^2+^ supplementation could be a possible therapeutic option for PD patients as currently there is no cure for PD. This is also important as the current treatments only rely on dopamine replacement therapy, which temporally alleviates the motor symptoms without stopping the neurodegeneration of DA neurons.

TRPM7 is an intrinsic ion channel that is central in modulating Mg^2+^ homeostasis. Cells where TRPM7 is downregulated had lower-than-normal levels of intracellular Mg^2+^ and Mg^2+^ supplementation to the culture media rescued cell growth and viability [41]. Our results also indicate that MPTP and MPP^+^ treatments significantly decrease TRPM7 levels and TRPM7 functions. Similarly, a decrease in TRPM7 expression was observed in PD samples. This decrease in TRPM7 expression was not a generalized effect, since actin levels were not decreased upon neurotoxin treatment. Moreover, our data also suggest that Mg^2+^ supplementation restored the expression of TRPM7, which indicate that TRPM7 alone could contribute to cell survival. However, increasing extracellular Mg^2+^ to 10 mM also inhibited TRPM7 currents, which suggest that a set-point of Mg^2+^ homeostasis is essential for neuronal survival. Importantly, knockdown of TRPM7 abolished the effect of Mg^2+^-induced protection and inhibition of caspase 3 activation was observed in cells treated with siTRPM7 and Mg^2+^. In contrast, overexpression of TRPM7 restored TRPM7 currents, inhibited the caspase activity, and promoted cell survival. Similar to these results, the protective effect of Mg^2+^ on cell death was also inhibited by the loss of TRPM7 expression and overexpression of TRPM7 showed protection against neurotoxins even at lower concentration of Mg^2+^. Based on these findings, we postulated that the decrease in TRPM7 levels upon MPTP and MPP^+^ treatment would contribute to altered Mg^2+^ homeostasis thereby leading to cell death. These results are important as even though expression of other Mg^2+^ transporters including TRPM6 and SLC41A1 are present they were unable to overcome the loss of TRPM7. Although the mechanism as to how TRPM7 protects dopaminergic neurons is not fully established, it could inhibit ROS formation by maintaining appropriate Mg^2+^ levels and inhibit oxidative stress [13]. Similarly, Mg^2+^ has been shown to reduce the presynaptic release of glutamate which could also prevent neurodegeneration by blocking the glutamatergic NMDA receptors in DA neurons [42].

Besides Mg^2+^, another study also reported that Ca^2+^ through TRPM7 plays a critical role in ROS-mediated cortical neurons death [43]. Our previous studies have also indicated that another member of the TRPM family “TRPM2” plays a key role in ROS-mediated dopaminergic neurodegeneration, which was due to an increase in Ca^2+^ influx [44]. Interestingly, TRPM7 and TRPM2 expressions in cortical neurons are coordinated [45]. Interestingly, we have previously shown that in MPTP/MPP^+^ models, TRPM2 expression is increased [44]; whereas, here, we observed that TRPM7 expression is decreased in dopaminergic neurons that are treated with the neurotoxins. We postulate that the anoxia-induced Ca^2+^ influx is perhaps carried by TRPM2 and/or TRPM2/TRPM7 heteromers that leads to neurodegeneration. In contrast, the protective Mg^2+^ influx is carried solely through TRPM7. One another interesting finding that we have observed was that neurotoxin treatment not only decreased TRPM7 function but also its expression was decreased. Although the mechanism is not well known, Mg^2+^ has been shown to modulate TRPM6/7 expression. In addition, the 5′ promoter region of TRPM7 has many binding sites for Ca^2+^-dependent transcription factors, including NFkB, and as TRPM7 also brings Ca^2+^, it could decrease TRPM7 expression, by inhibiting NFkB activation; however, more research is needed to fully evaluate this aspect.

Low Mg^2+^ concentrations in the brain tissues of PD patients have previously been reported [46, 47]. Similarly, Mg^2+^ concentration in the cerebrospinal fluid of PD patients decreased with the duration and severity of the disease [11]. However, circulating Mg^2+^ levels in PD etiology remain controversial, a study indicated PD patients tendend towards elevated circulating magnesium levels [48]. Epidemiological results support the possibility that mutations in genes relevant to magnesium homeostasis would alter PD risk [16]. Also, mutations in TRPM7 have been reported in some familial PD patients [21]. Although we did not look at the expression and function of these TRPM7 mutants, it could be suggested that certain TRPM7 mutations in PD patients might decrease its expression and/or function thereby inducing neuronal loss. Moreover, the TRPM7 channel plays an important role in cellular Mg^2+^ homeostasis [41], and the dysfunction of cellular Mg^2+^ transport could be a possible cause of PD. TRP channels have been shown to also express on synaptic vesicles, thus TRPM7 may also contribute to maintaining ion homeostasis in synaptic vesicles as defects in vesicle trafficking are proposed to play a major role in PD. Our study establishes the significance of TRPM7 in PD by using a combination of live-cell assays and biochemical and electrophysiological approaches including protein expression and channel activity. Our data suggest that TRPM7 expression and function regulate Mg^2+^ homeostasis and contribute to the survival of DA neurons. However, TRPM7 is permeable to ions other than Mg^2+^, and because it also has a kinase domain that can be cleaved and migrate to the nucleus to alter the epigenome or gene transcription [49, 50], which could also indirectly contribute towards cell survival. However, at present, we cannot rule out these other possibilities and further research is needed to confirm these hypotheses. Nonetheless, our results are important as we show for the first time that loss of TRPM7 might be the cause of neurodegeneration and Mg^2+^ supplementation that prevents the loss of DA neurons could be due to the restoration of TRPM7 expression and function.

## Electronic Supplementary Material

ESM 1 (PDF 67 kb)
