# Supplementary material for: Magnesium-Induced Cell Survival Is Dependent on TRPM7 Expression and Function
Source: Mol Neurobiol. 2019 Aug 8;57(1):528–38. doi: 10.1007/s12035-019-01713-7 (PMC6968994; doi:10.1007/s12035-019-01713-7)
Supplement: Supplementary file 1 — (PDF 67 kb) [file 12035_2019_1713_MOESM1_ESM.pdf]

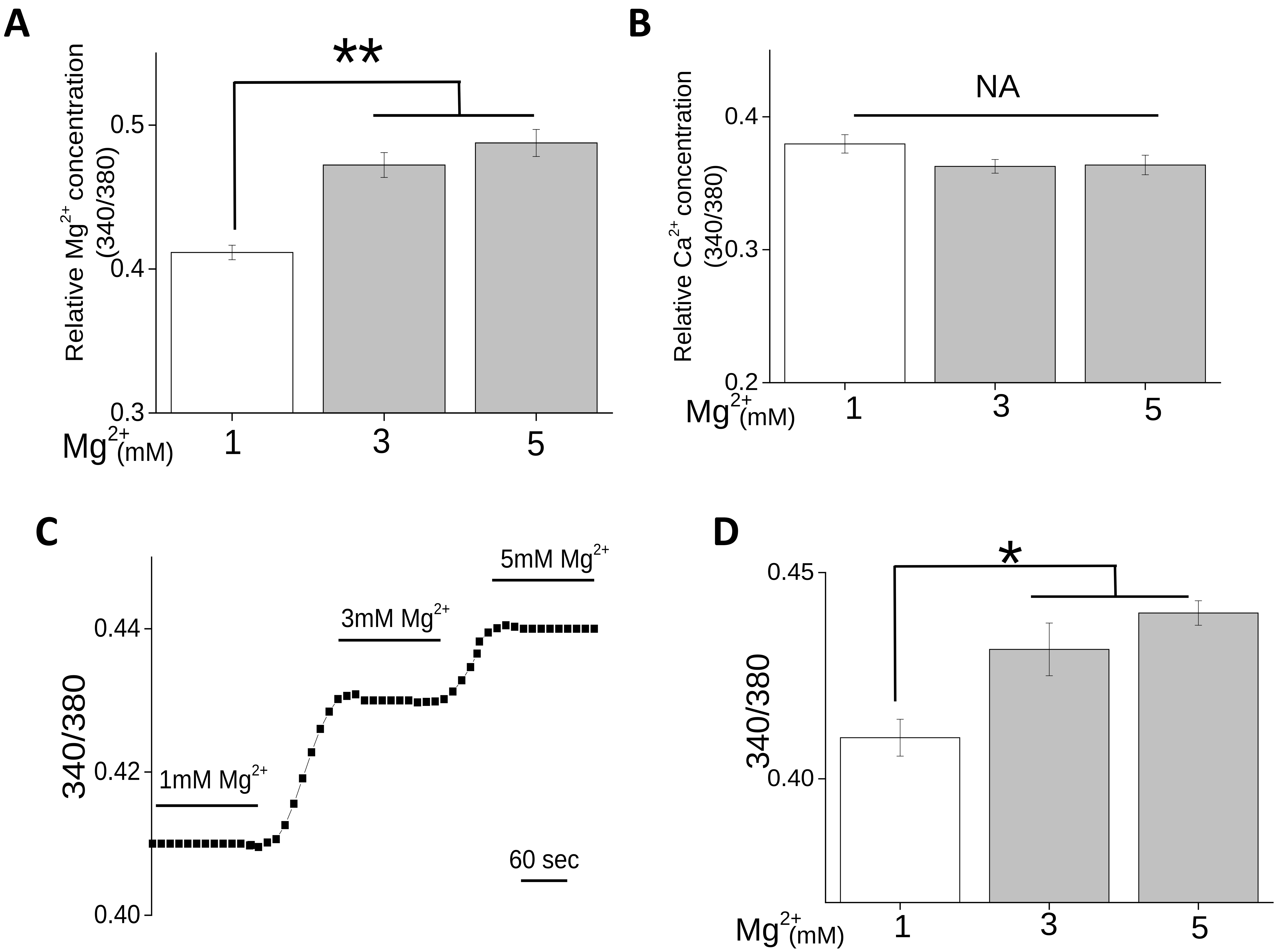

Supplemental Figure1. Bar diagram showing relative basal intracellular Mg^{2+} (**A**) and Ca $^{2+}$ (**B**) concentration under different Mg^{2+} environment culture for 24h in SH-SY5Y cells. (***p<0.01; One way ANOVA) (**C**) Mg^{2+} imaging was performed using mag-fura in SH-SY5Y cells. Bath application of Mg^{2+} induces Mg^{2+} influx and analog plots of the fluorescence ratio (340/380) are shown. (**D**) Quantification (mean ± SE) of Mg^{2+} concentration. * indicate significance (p <0.05).
